# The role and research progress of FGF21 in breast cancer: a review

**DOI:** 10.3389/fonc.2026.1792848

**Published:** 2026-03-11

**Authors:** Jiali Chen, Chengyao Chang, Xuan Yang

**Affiliations:** 1 Shanxi Provincial People’s Hospital Affiliated to Shanxi Medical University, Taiyuan, China; 2 Department of Breast Surgery, Shanxi Provincial People’s Hospital, Taiyuan, China

**Keywords:** breast cancer, FGF21, metabolic reprogramming, signaling pathway, therapeutic target, tumor microenvironment

## Abstract

**Purpose:**

To investigate the molecular mechanisms, functional controversies, and clinical significance of Fibroblast Growth Factor 21 (FGF21) in the context of breast cancer.

**Methods:**

A comprehensive literature search was conducted across databases including PubMed and CNKI to summarize recent advances regarding FGF21 in metabolic reprogramming and immune microenvironment remodeling.

**Results:**

FGF21 primarily serves as a pro-tumorigenic factor in breast cancer through several key mechanisms: (1) Metabolic Reprogramming: It activates the ERK1/2-SENP2 axis to upregulate CD36, which enhances fatty acid oxidation to fuel tumor metastasis. (2) Immune Evasion: It induces CD8^+^ T cell exhaustion by persistently activating intracellular cholesterol synthesis pathways. (3) Anti-apoptosis: It enhances chemoresistance by activating signaling pathways such as STAT3. Clinically, elevated FGF21 levels are significantly correlated with disease progression and poor prognosis, particularly in patients with metabolic comorbidities.

**Conclusion:**

FGF21 acts as a pivotal bridge connecting systemic metabolism with local tumor behavior. Future research should focus on developing precision intervention strategies that preserve its systemic metabolic benefits while selectively blocking its local oncogenic effects.

## 1 Introduction

Breast cancer remains one of the most prevalent malignancies among women, with its incidence and mortality rates increasing annually, posing a significant threat to global female health (1). Fibroblast Growth Factor 21 (FGF21) is a distinct endocrine growth factor that structurally belongs to the FGF19 subfamily of the FGF superfamily (2). Distinguished from canonical FGF members by the absence of a typical heparin-binding domain, FGF21 can bypass the extracellular matrix to enter the systemic circulation as a hormone-like factor, thereby exerting systemic regulatory influence (3). Primarily synthesized and secreted by the liver, adipose tissue, and pancreas, FGF21 serves as a core regulator in maintaining metabolic homeostasis (4). As a critical metabolic factor, FGF21 enhances insulin sensitivity, promotes glucose uptake, and modulates fatty acid oxidation and ketogenesis to maintain energy balance (5). Nevertheless, accumulating evidence highlights the pivotal role of FGF21 in the initiation and progression of various malignancies (6–8), where it promotes cell proliferation, inhibits apoptosis, and reshapes the tumor microenvironment.

In the field of oncology, particularly regarding breast cancer, the expression patterns and biological functions of FGF21 are remarkably complex. While some researchers suggest a pro-tumorigenic role for FGF21, others propose its potential as a tumor suppressor. Although expressed at low levels in normal breast tissue, FGF21 is frequently upregulated across multiple breast cancer subtypes, correlating strongly with advanced disease stages and unfavorable prognosis. This cancer-specific expression profile underscores the potential importance of FGF21 in breast cancer pathogenesis. Consequently, a comprehensive investigation into the biological functions and specific mechanisms of FGF21 in breast cancer is essential for elucidating its molecular basis, developing innovative therapeutic strategies, and identifying novel biomarkers for early diagnosis and prognostic assessment.

## 2 Literature search strategy

While the body of research concerning the relationship between FGF21 and breast cancer has expanded steadily, current findings remain inconsistent. To address this, a comprehensive literature search was conducted across major databases, including PubMed, Web of Science, CNKI, and Wanfang, using keywords such as “FGF21,” “breast cancer,” “malignant tumor,” and “tumor microenvironment”. The literature considered spanned from the inception of the respective databases up to December 2025. Inclusion criteria were strictly limited to peer-reviewed original research articles, systematic reviews, and meta-analyses published in English or Chinese. To ensure the reliability and reproducibility of the synthesized data, non-peer-reviewed preprints, conference abstracts lacking full datasets, and articles in other languages were excluded.

Building upon this search, the present study provides a systematic review of the roles of the FGF21 protein and its receptors in breast cancer. This review focuses on the underlying molecular mechanisms, associated signaling pathways, and the potential for clinical application, aiming to establish a robust theoretical foundation for future research and therapeutic development. The basic biological characteristics and functional comparisons of FGF21 versus canonical FGFs are summarized in The following **Table 1**.

**Table 1:** Basic biological characteristics and functions of FGF21 protein.

Characteristic	FGF21	Canonical FGFs (e.g., bFGF)
Structural Features	Lack of a typical heparin-binding domain	Possess a typical heparin-binding domain
Mode of Action	Endocrine factor	Autocrine/Paracrine factor
Primary Source	Liver and adipose tissue	Widely expressed across various tissues
Specific Receptor	FGFR1-KLB complex	FGFRs (without a requirement for KLB)
Main Physiological Function	Metabolic regulation (glucose and lipid metabolism)	Cell proliferation, differentiation, and angiogenesis
Role in Breast Cancer	Dual role (controversial pro-tumorigenic vs anti-tumorigenic effects)	Established pro-tumorigenic role

## 3 Composition and function of the FGF21 signaling pathway

The FGF21 Receptor Complex: This complex comprises FGFR1c (Fibroblast Growth Factor Receptor 1c) and β-Klotho (9). FGFR1c serves as the primary receptor for FGF21, while β-Klotho acts as an essential co-receptor to form the functional receptor complex (10). Through this FGFR1c/β-Klotho complex, FGF21 preferentially activates the MAPK pathway (11). These receptors are ubiquitously expressed in tumor cells and frequently exhibit aberrant activation in various cancers (12).

The dysregulation of FGF expression drives oncogenic FGFR signaling, which in turn activates downstream pathways—including MAPK-ERK, PI3K-AKT, and JAK-STAT—to regulate tumor cell proliferation, differentiation, survival, and migration (13). The FGF/FGFR signaling pathway plays a critical role in embryonic development, organogenesis, metabolic homeostasis, tissue repair, regeneration, and tumor angiogenesis (14). In breast cancer progression, FGFRs along with their numerous FGF ligands and signaling partners are frequently altered, contributing to therapeutic resistance (15).

Analysis reveals that the complexity of FGF21-related signaling resides in the “diversity” and “cell-type specificity” of its downstream effects. While it primarily regulates energy balance in metabolic organs, it shifts to prioritize pro-survival signals such as MAPK/ERK and PI3K/AKT within the tumor microenvironment. Notably, research has identified an aberrant isoform switching of FGFR1 splice variants in tumor cells (9), which may be a key factor underlying the divergent FGF21 signaling outputs across different breast cancer subtypes. This suggests that understanding FGF21 function necessitates consideration of its specific receptor context, as a simplistic “ligand-function” model is insufficient to explain its intricate behavior in cancer.

## 4 Molecular mechanisms of FGF21-mediated breast cancer progression

### 4.1 Metabolic reprogramming mechanisms

Under physiological conditions, FGF21 promotes fatty acid oxidation and ketogenesis to maintain energy homeostasis in response to metabolic stresses such as starvation. A prevailing trend in the field is to more closely link metabolic reprogramming with the malignant behavior of tumors. The rapid proliferation of breast cancer cells requires a substantial supply of energy and nutrients (16), and FGF21 plays a critical role in this process. By modulating metabolic pathways—such as promoting glycolysis and fatty acid oxidation—FGF21 satisfies the high energy demands required for rapid tumor cell proliferation. Secreted by hepatocytes, FGF21 synergizes with PPARα to regulate hepatic ketogenesis, gluconeogenesis, and fatty acid oxidation (17). FGF21 upregulates PGC-1α, a transcriptional coactivator of PPARα, thereby modulating the expression of gluconeogenic genes (18, 19). Furthermore, research suggests that FGF21 directly participates in gluconeogenesis by modulating glucose-6-phosphatase (G6Pase) and phosphoenolpyruvate carboxykinase (PEPCK) (20). Given that high levels of fructose-1,6-bisphosphatase (FBPase) are positively correlated with breast cancer metastasis, gluconeogenesis may represent a potential metabolic therapeutic target for breast cancer patients (21). Additionally, FGF21 stimulates the expression of adiponectin, which plays a vital role in maintaining glucose and lipid metabolism and homeostasis (22, 23). This metabolic reprogramming not only facilitates the survival and proliferation of cancer cells but may also enhance their anti-apoptotic capacity, thereby further exacerbating the malignant progression of breast cancer.

**Figure 1:**
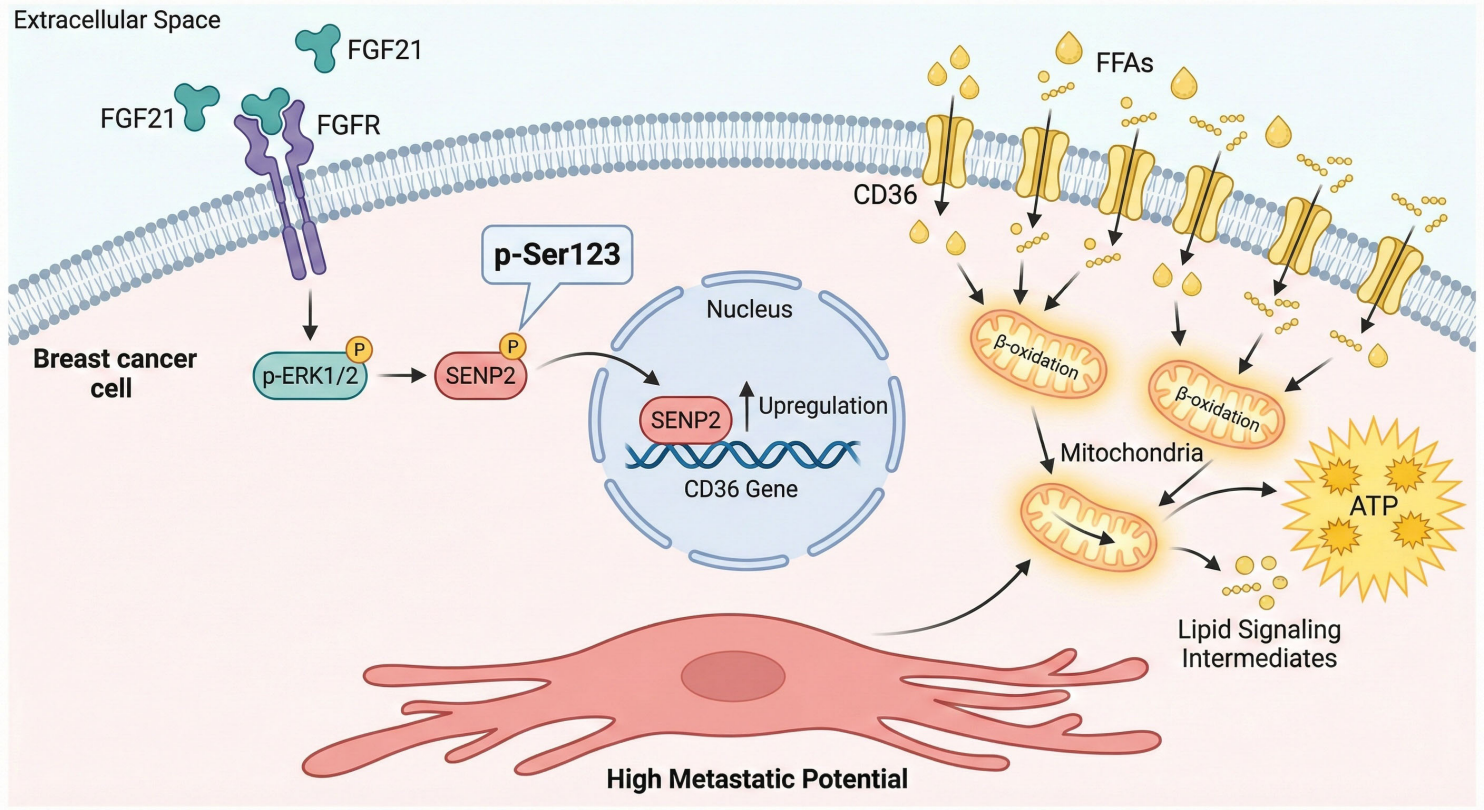
FGF21 regulates metabolic reprogramming.

FGF21 fulfills the energy and biosynthetic requirements for the rapid proliferation of tumor cells by activating lipid metabolic pathways. Research has demonstrated that FGF21 promotes the activation of SENP2 through ERK1/2-mediated phosphorylation at Serine 123 (Ser123). Subsequently, activated SENP2 upregulates the expression of the fatty acid transport receptor CD36. As a pivotal fatty acid transporter (24), increased CD36 expression enhances the uptake of free fatty acids (FFAs) by tumor cells (25), providing ample substrates for β-oxidation (26). This lipid metabolic reprogramming equips breast cancer cells with the bioenergetic profile necessary for high metastatic potential: on one hand, augmented fatty acid oxidation ensures a robust supply of ATP to support the energy demands of cell migration and invasion (27); on the other hand, lipid metabolic intermediates function as signaling molecules that can activate multiple pro-metastatic pathways (28). The FGF21-mediated upregulation of CD36 enhances fatty acid uptake, which serves not only as a fuel source but also generates metabolites (e.g., lipid signaling molecules) that directly activate various pro-metastatic pathways, thereby establishing a “metabolic-signaling” positive feedback loop (**Figure 1**).

### 4.2 Mechanisms of immunosuppressive microenvironment formation

Cancer immunity and immune tolerance within the tumor microenvironment (TME) are regulated by the complex interactions between neoplastic cells and immune cells (29). CD8+ T cells, natural killer (NK) cells, and natural killer T (NKT) cells serve as the primary immune effector cells responsible for tumor clearance (30). Conversely, myeloid-derived suppressor cells (MDSCs), M2-type tumor-associated macrophages (M2-TAMs), and regulatory T (Treg) cells act as immune-modulating cells that facilitate immune evasion and promote tumor growth (31–33). The activation of FGFR1 in both breast cancer cells and stromal cells induces the production of the chemokine CX3CL1 (fractalkine), which subsequently recruits CX3CR1-expressing macrophages and monocytes into the TME (34). Upon exposure to factors such as FGF21 within the TME, these recruited macrophages may undergo polarization toward the M2 phenotype (35, 36), characterized by potent immunosuppressive and pro-tumorigenic properties (37).

Within the tumor microenvironment (TME), the anti-tumor efficacy of CD8^+^ cytotoxic T cells is profoundly dependent on their metabolic fitness. Under physiological conditions, the expansion and effector functions of activated T cells necessitate substantial lipid synthesis to provide structural membrane components and signaling precursors, with cholesterol uptake and *de novo* biosynthesis being under stringent regulation. However, in the breast cancer microenvironment, tumor cells frequently outcompete immune cells for essential nutrients, driving T cells into a state of metabolic dysfunction.

Research by Jun Gui and colleagues has elucidated the detailed molecular mechanisms by which FGF21 mediates tumor immune evasion. This study identified that tumor-secreted FGF21 acts as an immunosuppressive factor by specifically binding to the FGFR1-KLB receptor complex on the surface of tumor-infiltrating CD8^+^ T cells to trigger downstream signaling. Mechanistically, the formation of the FGF21-FGFR1-KLB heterotrimer induces a phosphorylation cascade that constitutively activates the AKT-mTORC1-SREBP1 signaling axis. The hyperactivation of this pathway promotes the nuclear translocation of the SREBP1 transcription factor, subsequently upregulating genes involved in cholesterol biosynthesis and leading to intracellular cholesterol overload in CD8^+^ T cells.

**Figure 2:**
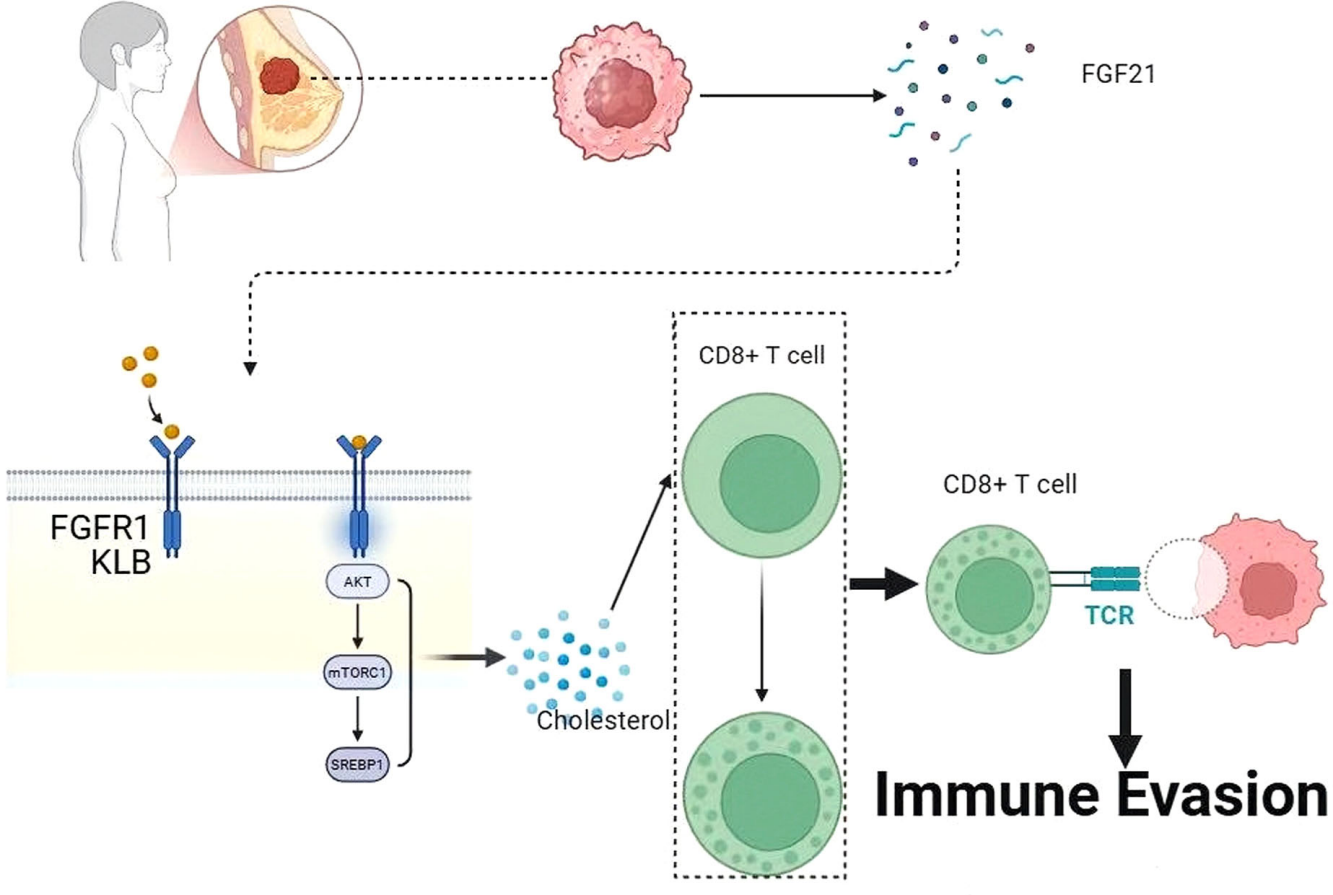
Tumor-derived FGF21 mediates immune evasion by modulating CD8+T cells.

The accumulation of excessive cholesterol alters cell membrane fluidity and interferes with the assembly of the immunological synapse, ultimately resulting in CD8^+^ T cell exhaustion. This state is characterized by a marked reduction in the secretion of effector cytokines, such as IFN-γ and TNF-α, and a significant decline in tumor-killing capacity (38). These findings provide a molecular explanation for why patients often exhibit poor clinical outcomes despite the presence of T cell infiltration in breast cancer tissues with high FGF21 expression. This discovery refines the traditional view of FGF21 as a purely metabolic benefit factor, establishing its novel role as a potent “immunometabolic checkpoint”. This immunosuppressive function operates independently of the classical metabolic regulatory roles of FGF21, thereby expanding the understanding of its pleiotropic effects. Furthermore, the study demonstrated that blocking this pathway with an FGF21-neutralizing antibody effectively restores the anti-tumor activity of CD8^+^ T cells and exhibits synergistic therapeutic effects when combined with PD-1 blockade (**Figure 2**).

### 4.3 Mechanisms of cell survival and anti-apoptosis

FGF21 typically functions as a cytoprotective factor, precluding cell death under conditions of metabolic stress. Research by Chen et al. has elucidated the mechanisms through which FGF21 confers anti-apoptotic protection to breast cancer cells. In the setting of non-alcoholic fatty liver disease (NAFLD), elevated circulating FGF21 levels accumulate within breast tumor tissues, subsequently activating the STAT3 and Akt/FoxO1 signaling pathways (39). As a critical transcriptional regulator, STAT3 activation upregulates the expression of several anti-apoptotic proteins, such as Bcl-2 and Bcl-xL (40, 41). Meanwhile, the Akt/FoxO1 pathway enhances cell survival by suppressing the activity of pro-apoptotic factors (42).

**Figure 3:**
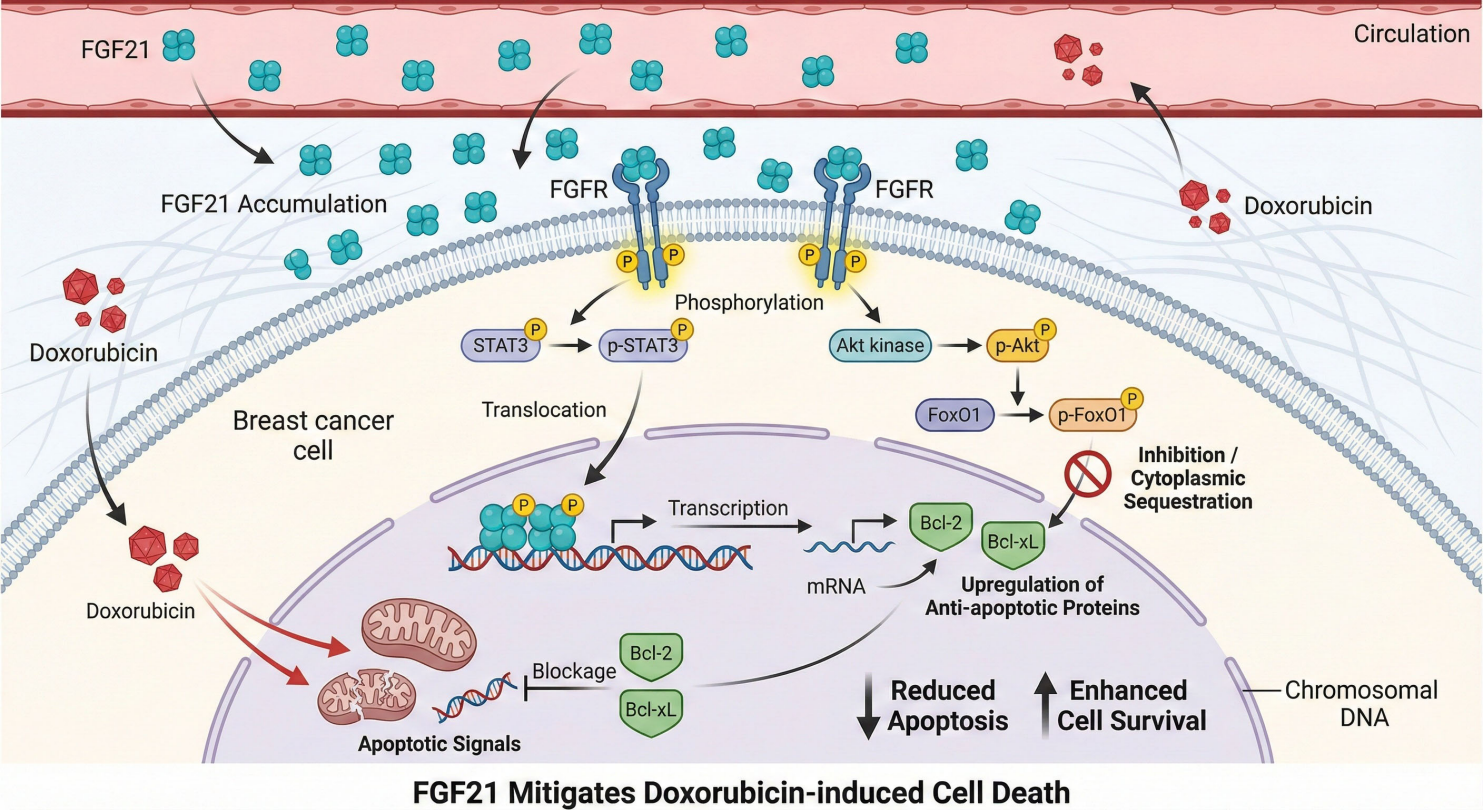
FGF21 induces anti-apoptosis in breast cancer cells.

This mechanism is particularly significant in the context of chemotherapy. Investigations have shown that FGF21 treatment substantially attenuates breast cancer cell death induced by chemotherapeutic agents such as doxorubicin, providing a molecular rationale for the diminished therapeutic efficacy observed in patients with metabolic abnormalities, including obesity and NAFLD. Analyses of clinical specimens further corroborate these findings, revealing that FGF21 overexpression in breast cancer tissues is closely linked to an unfavorable prognosis, underscoring its potential utility as a prognostic biomarker (**Figure 3**).

**Figure 4:**
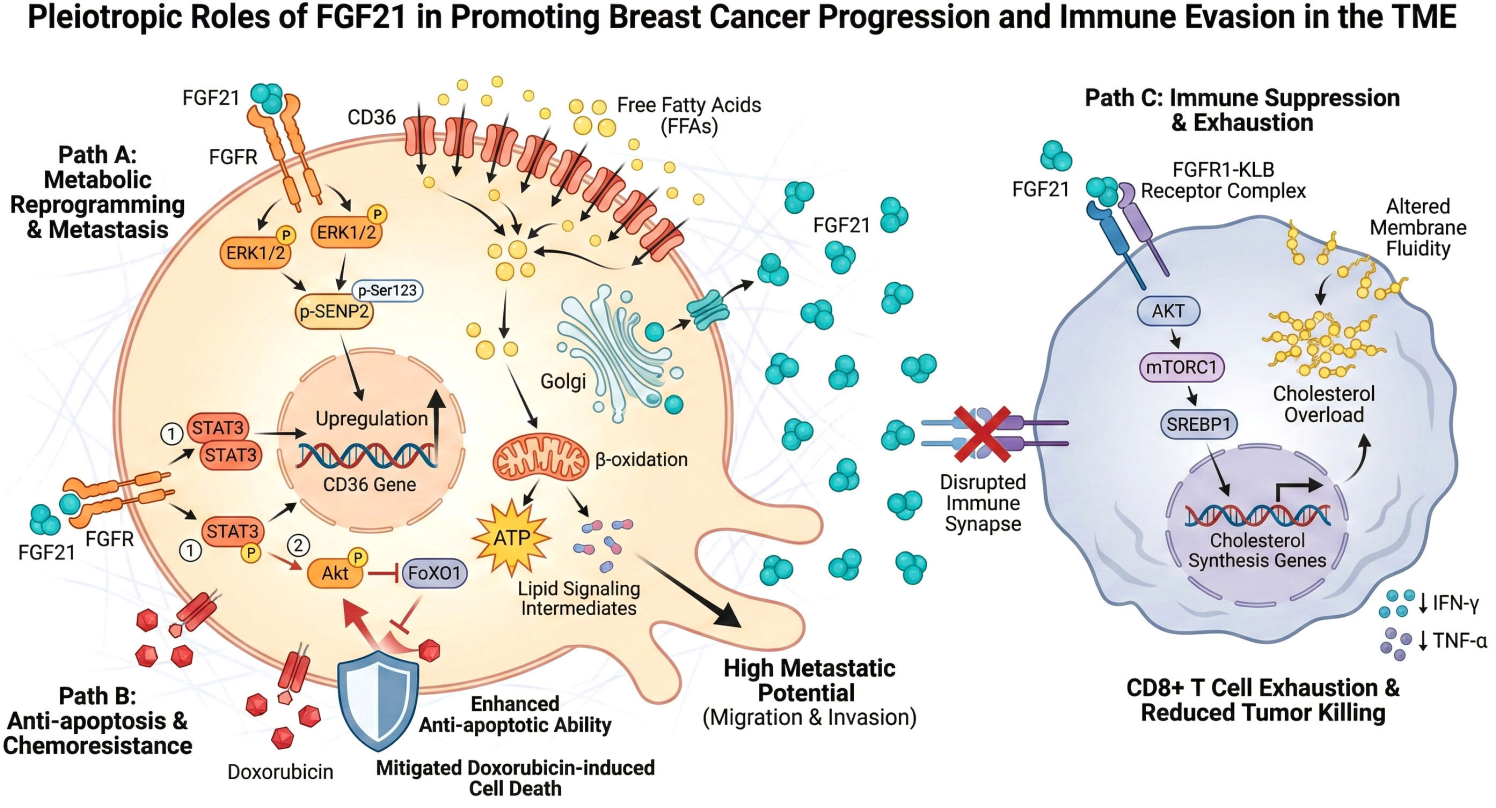
Molecular mechanisms by which FGF21 mediates breast cancer progression.

A comprehensive overview of these pleiotropic molecular mechanisms—encompassing metabolic reprogramming, immune evasion, and anti-apoptosis—is illustrated in **Figure 4**.

## 5 Research progress of FGF21 in breast cancer

### 5.1 Pro-tumorigenic effects

Numerous preclinical studies have confirmed that FGF21 is aberrantly overexpressed in the breast cancer microenvironment and promotes tumor progression through multiple mechanisms. The study by Chen et al. was the first to systematically reveal the pivotal role of FGF21 as a key link between non-alcoholic fatty liver disease (NAFLD) and breast cancer progression. By connecting systemic disease with tumor progression through a distinct molecular bridge, this research provides a mechanistic explanation for the poor prognosis observed in patients with metabolic comorbidities. Studies indicate that under NAFLD conditions, hepatic secretion of FGF21 increases significantly; these circulating FGF21 molecules are transported via the bloodstream to breast tumor tissues, where they accumulate and accelerate tumor growth (39).

Furthermore, the role of FGF21 in obesity-associated breast cancer warrants special attention, particularly given that adipose tissue serves as a primary physiological source of FGF21 alongside the liver. In the context of obesity, the pro-tumorigenic effects of FGF21 are profoundly amplified through both systemic and local microenvironmental mechanisms. Systemically, obesity-induced metabolic stress—such as non-alcoholic fatty liver disease (NAFLD)—triggers the hepatic overproduction of endocrine FGF21, which travels via the circulation to enhance tumor cell anti-apoptotic capacity and chemoresistance (39).

More importantly, locally within the breast, the expanded adipose mass plays a direct oncogenic role. Recent evidence reveals that cancer-associated adipocytes (CAAs) residing in the tumor microenvironment act as a robust source of autocrine and paracrine FGF21. This locally secreted FGF21 activates the FGFR1/KLB-p38 signaling axis, upregulating adipose triglyceride lipase (ATGL) and fiercely driving lipolysis. The consequent massive release of free fatty acids (FFAs) into the microenvironment leads to severe lipid peroxidation and mitochondrial damage in tumor-infiltrating CD8+ T cells, ultimately resulting in irreversible T cell exhaustion and robust immune evasion (38). Therefore, in obesity-associated breast cancer, adipose tissue-derived FGF21 acts as a critical local driver that bridges altered lipid metabolism with profound tumor immunosuppression.

In animal models, the exogenous administration of recombinant FGF21 leads to a marked increase in tumor volume and weight, whereas the specific knockout of the FGF21 gene effectively blocks the tumor-promoting effects induced by a high-fat diet. At the molecular level, FGF21 promotes tumor survival by enhancing the anti-apoptotic capacity of breast cancer cells. Research has found that FGF21 treatment activates the STAT3 and Akt/FoxO1 signaling pathways, thereby strengthening the ability of cancer cells to resist apoptosis—specifically by mitigating cell death induced by chemotherapeutic agents such as doxorubicin. This discovery provides a mechanistic rationale for the association between metabolic abnormalities (such as obesity and NAFLD) and the poor prognosis of breast cancer, while also offering a theoretical foundation for targeted interventions.

The research led by Cheng’s team further elucidated the mechanisms by which FGF21 influences breast cancer metastasis. In Luminal-type and HER2-positive breast cancers, serum FGF21 levels are significantly and positively correlated with tumor metastatic potential. However, while current evidence strongly supports the pro-tumorigenic role of FGF21 in Luminal and HER2-positive environments, there is a distinct lack of data regarding its specific effects in other molecular subtypes, particularly the highly aggressive triple-negative breast cancer (TNBC). Mapping whether FGF21’s signaling cascades are conserved or functionally divergent in TNBC remains a critical gap in current tumor heterogeneity research. FGF21 activates the SENP2 protein via ERK1/2-mediated phosphorylation (at the Ser123 site), which in turn upregulates the expression of the fatty acid transport receptor CD36 (43). This ultimately enhances the lipid metabolism capacity of breast cancer cells, providing the necessary energy support for metastasis. This finding directly links FGF21 signaling with tumor metabolic reprogramming, explaining the metabolic basis for enhanced invasion and metastasis. It is suggested that this work directly couples FGF21 signaling with the cellular metabolic engine, clarifying the complete “Signal to Metabolism to Function” chain and explaining how tumor cells acquire the energy required for metastasis.

Research by Ranuncolo et al. demonstrated that in treatment-naive breast cancer patients, serum FGF21 levels are significantly higher than those in healthy controls. Notably, regarding disease progression, elevated plasma FGF21 is detectable even in early-stage breast cancer, where it is already associated with systemic immune dysregulation and the downregulation of immune checkpoint molecules. As the disease advances to metastatic stages, particularly in Luminal and HER2-positive subtypes, these levels rise further and positively correlate with metastatic potential. However, it should be noted that current evidence primarily relies on cross-sectional comparisons, and there is a distinct lack of longitudinal tracking data within the same cohort to definitively map these dynamic changes from early to advanced stages. Furthermore, high FGF21 levels (>130.64 pg/mL) serve as an independent risk factor for shortened overall survival. Multivariate analysis confirmed that this negative correlation persists even after adjusting for factors such as tumor stage and age, highlighting its potential as an independent prognostic biomarker. Recent clinical evaluations further corroborate its diagnostic value. A study by Arslan et al. demonstrated that serum FGF21 levels were significantly elevated in breast cancer patients compared to healthy controls, and established that an FGF21 cutoff value of 86.83 pg/mL could serve as a novel diagnostic biomarker with 68% sensitivity and 52% specificity (44). Nevertheless, the clinical utility of FGF21 as a standalone biomarker is limited by confounding factors. Because metabolic comorbidities such as obesity, type 2 diabetes, and non-alcoholic fatty liver disease (NAFLD) can independently and significantly elevate systemic FGF21 levels, the specificity of FGF21 for breast cancer detection may be diluted. Therefore, FGF21 may be best utilized in the clinic not as a broad screening tool, but alongside a panel of markers to assess prognosis specifically in patients with metabolism-associated breast cancer. This study also noted that the elevation of FGF21 does not fully parallel the degree of obesity, suggesting that its upregulation may be primarily driven by the tumor itself or the systemic stress state it induces (45).

In summary, current research collectively portrays FGF21 as a “metabolic amplifier” in the context of cancer promotion. Rather than acting as a traditional oncogenic driver, it is significantly activated when the body or local microenvironment is under metabolic stress (such as NAFLD or obesity). It integrates multiple signals to convert abundant metabolites, such as lipids, into the “fuel” and “instructions” required for malignant behavior. This understanding revolutionizes the perception of FGF21 function: its pro-tumorigenic effect is essentially the “hijacking” and “amplification” of its physiological metabolic regulatory role within a pathological tumor context. Consequently, elevated circulating FGF21 levels may be more than a simple disease marker; they signify that the body has entered a specific stage where metabolic dysregulation drives rapid tumor progression. This provides a critical theoretical basis for implementing precise metabolic intervention therapies for breast cancer patients with metabolic abnormalities.

### 5.2 Tumor suppressive effects

In contrast to the aforementioned pro-tumorigenic findings, several studies have documented potential tumor-suppressive effects of FGF21 in breast cancer. Research by Luo et al. demonstrated that elevated systemic levels of FGF21—such as those observed in liver-specific FGFR4-deficient models—can effectively delay the initiation and progression of mammary tumors (46). Using an MMTV-TGFα transgenic mouse model with a global knockout of FGFR4, the study found that FGFR4 deficiency led to a significant upregulation of hepatic FGF21 expression and secretion, accompanied by reduced tumor incidence, delayed tumor progression, and improved host survival. Mechanistic analysis indicated that this tumor-suppressive effect did not stem from FGF21’s direct action on tumor cells, but rather through its classic endocrine pathway: elevated circulating FGF21 acts on adipocytes in the breast microenvironment that express the KLB-FGFR1 complex. This promotes the secretion of adiponectin, which has known tumor-suppressive activity, and downregulates pro-tumorigenic factors such as IGF-1 and TIMP1. This systemic and microenvironmental metabolic remodeling, driven by FGF21, collectively creates a microenvironment that inhibits tumor development.

However, it must be noted that the global gene knockout model used in this study may not accurately reflect the spontaneous metabolic evolution and local autocrine signaling in human breast cancer. Therefore, although this systemic tumor-suppressive effect is theoretically significant, its physiological relevance requires further validation in more translationally relevant models, such as conditional knockout or humanized models.

Specifically, FGF21 promotes the secretion of the tumor-suppressive adipokine adiponectin (47), inhibits pro-tumorigenic factors such as IGF-1 (48) and TIMP1 (49), and induces metabolic reprogramming in adipocytes. This reprogramming includes the enhancement of fatty acid oxidation, the inhibition of glycolysis, and a reduction in the expression of the essential metabolic enzyme NAMPT, collectively establishing a microenvironment hostile to tumor cell proliferation and survival (46).

Reconciling these contradictory findings requires the consideration of several critical factors: FGF21 Source: The functional outcome may be dictated by the source of the protein, as tumor-derived autocrine FGF21 and liver-derived endocrine FGF21 likely exert distinct biological effects. Molecular Subtypes: Different breast cancer subtypes (e.g., Luminal versus triple-negative) may exhibit differential responsiveness to FGF21 signaling. Metabolic Status: The specific metabolic context of the tumor microenvironment, such as local lipid concentrations, may modulate the functional direction of FGF21 action.

The evidence indicates these seemingly contradictory findings underscore the inherent functional complexity of FGF21 rather than reflecting simple experimental discrepancies. Based on our comprehensive review, we propose a unified “systemic-local dichotomy” framework to reconcile this paradox: endocrine FGF21 derived from the liver indirectly creates a “soil” inhospitable to tumor growth by optimizing the systemic metabolic milieu (for instance, by stimulating adiponectin secretion); conversely, autocrine FGF21 produced locally by the tumor acts directly upon the cancer cells and their microenvironment, serving as a “seed” that drives malignant progression. Consequently, the ultimate biological manifestation of FGF21 represents the “net result” of integrated signaling across various spatial scales within the organism. In the context of most advanced breast cancers, local pro-oncogenic signaling typically predominates, resulting in an overall tumor-promoting phenotype for FGF21.

Synthesizing the available evidence, FGF21 predominantly functions as a tumor-promoting factor in breast cancer, operating primarily through two core mechanisms: the induction of metabolic reprogramming and the establishment of an immunosuppressive microenvironment. The tumor-suppressive effects reported in certain studies may arise from specific experimental parameters or dose-dependent responses, and their physiological significance necessitates further rigorous verification.

To provide a clearer comparison of these divergent findings, **Table 2** summarizes the contrasting pro-tumorigenic and tumor-suppressive mechanisms of FGF21, highlighting key studies, their experimental contexts, and core pathways.

**Table 2:** Summary of the dual roles and mechanisms of FGF21 in breast cancer.

Role	Key study and model	FGF21 axis (source → target)	Key pathway	Core mechanism and clinical effect
Pro-tumorigenic	Cheng et al (43). (Luminal/HER2 *in vitro* & *in vivo* models)	Autocrine/Exogenous → Breast cancer cells	ERK1/2 → SENP2 → CD36 ↑	Enhances fatty acid uptake and oxidation; promotes tumor metastasis.
	Sui et al (39). (NAFLD-associated mouse models)	Hepatic (Endocrine) → Breast cancer cells	STAT3/Akt-FoxO1 → Bcl-2 ↑	Increases anti-apoptotic capacity; promotes growth and chemoresistance.
	Hu et al (38). (CAA & CD8+ T cell co-culture models)	Tumor-adjacent adipocytes → CD8+ T cells	FGFR1/KLB-AKT-mTORC1 hyperactivation	Induces CD8+ T cell lipid overload and exhaustion; promotes immune evasion.
Tumor-Suppressive	Luo et al (46). (FGFR4-deficient transgenic mice)	Hepatic (Endocrine) → Adipocytes	Adiponectin ↑/IGF-1 ↓	Remodels systemic/local metabolic microenvironment; delays tumorigenesis.

Furthermore, to provide a comprehensive landscape of both preclinical mechanisms and clinical observational data regarding FGF21 in breast cancer, **Table 3** summarizes the key findings across different study designs.

**Table 3:** Overview of key preclinical and clinical studies on FGF21 in breast cancer.

Experiment type	Key conclusions and findings	Author(s)
Clinical observational study (Prospective cohort)	High FGF21 expression at the time of diagnosis is significantly correlated with reduced survival rates; notably, serum FGF21 levels significantly decline following one year of adjuvant endocrine therapy	Akyol et al (45)
Case-control study (Cross-sectional analysis)	Serum FGF21 levels are markedly higher in breast cancer patients compared to healthy controls, serving as a highly sensitive diagnostic biomarker; elevated FGF21 also correlates with shortened overall survival (OS), identifying it as a potent prognostic indicator	Rapoport et al (34)
Case-control observational study	It provides a specific diagnostic cutoff value (86.83 pg/mL) with a sensitivity of 68% and a specificity of 52%	Arslan et al (44).
Diagnostic and prognostic study (Retrospective cohort)	Early-stage breast cancer patients exhibit significantly elevated plasma FGF21 alongside systemic immune dysregulation (e.g., widespread downregulation of checkpoint molecules). This elevation may be linked to the induction of the M2 macrophage phenotype	Ranuncolo et al (50).
Animal models, cell experiments, and clinical specimen analysis (Mechanistic and prognostic study)	Non-alcoholic fatty liver disease (NAFLD) promotes breast cancer progression by upregulating hepatic-derived FGF21 into the circulation. FGF21 subsequently activates the STAT3 and Akt/FoxO1 pathways, enhancing anti-apoptotic capacity and conferring chemoresistance	Sui et al (39)
Prospective observational study	In a one-year follow-up, FGF21 levels showed no statistically significant change (median shift from 68.57 pg/mL to 92.79 pg/mL, p=0.226) and demonstrated no correlation with changes in body composition parameters	Larrad-Sainz et al (51)
Prospective pilot study	In postmenopausal patients, serum FGF21 significantly decreased one month after a 60 mg denosumab injection and remained low at five months, suggesting that denosumab modulates glucose homeostasis via the RANKL/FGF21 axis	
Genetically modified mouse models (FGFR4 KO and TGFα transgenic)	FGFR4 deficiency is associated with reduced tumor incidence, delayed progression, and improved survival, all of which correlate with an compensatory increase in systemic FGF21 levels	Luo et al (46)
Serum adipokine array analysis	FGFR4-deficient mice exhibit a five-fold increase in serum FGF21 and elevated adiponectin, while pro-tumorigenic factors such as IGF-1 and TIMP1 are concurrently downregulated	
Quantitative PCR (qPCR) of liver tissues	Hepatic FGF21 expression increases by 2.5–4 times in FGFR4-deficient models, establishing FGF21 as a master regulator of systemic metabolic alterations	
Gene expression analysis of breast tissues (qPCR)	Elevated FGF21 orchestrates metabolic reprogramming in breast tissue—characterized by increased adiponectin, enhanced fatty acid oxidation, and suppressed glycolysis—thereby inhibiting tumor growth	
Tumor cell culture and NAMPT inhibition assays	The FGF21 downstream target NAMPT is highly expressed in tumor cells; pharmacological inhibition of NAMPT (via FK866) effectively reduces tumor cell proliferation and spheroid formation	
Patient serum ELISA detection	In Luminal and HER2-positive subtypes, serum FGF21 concentrations positively correlate with metastatic potential; concentrations exceeding 50 pg/mL identify a significantly higher risk of metastasis	Cheng et al (43)
Gene/protein expression analysis (Western Blot, qPCR)	FGF21 upregulates the fatty acid transporter CD36 and key metabolic genes (e.g., ACSL1, FA2H) via the ERK1/2 signaling pathway, thereby stimulating lipid metabolism to fuel tumor cells	Hu et al (38)
*In vitro* cell assays and *in vivo* mouse models	FGF21 triggers the AKT-mTORC1-SREBP1 signaling axis in CD8+ T cells, leading to cholesterol overload and subsequent T-cell exhaustion, a phenomenon particularly robust in breast cancer models	

## 6 Discussion

Current Limitations of Research: Ambiguous Functional Definition: Existing studies have failed to clearly distinguish between the systemic metabolic regulatory roles and the local pro-tumorigenic functions of FGF21.Neglect of Tumor Heterogeneity: There is a significant lack of differentiated research concerning how various breast cancer molecular subtypes respond to FGF21 signaling. Insufficient Translational Evidence: The field currently lacks large-scale clinical validation and specialized targeted therapeutic strategies. Future Directions and Strategic Goals Future research should transcend the binary “pro-cancer vs. anti-cancer” debate and instead focus on resolving the spatiotemporal-specific regulatory mechanisms of the FGF21 signaling network. Key priorities include: Source-Specific Modeling: Developing tools and models capable of distinguishing the different functional sources (e.g., autocrine vs. endocrine) of FGF21.Subtype-Specific Mapping: Precisely mapping the downstream signaling profiles of FGF21 within various molecular subtypes of breast cancer. Precision Intervention: Exploring strategies based on antibodies or small-molecule drugs to “promote the good while suppressing the evil”—specifically blocking tumor-promoting functions while preserving beneficial metabolic effects.

Translational Perspectives Future translational efforts should concentrate on the development of bispecific antibodies or conditional knockout strategies. These approaches aim to selectively block the local tumor-specific FGFR1-βKlotho signaling axis without compromising the systemic benefits of FGF21 on insulin sensitivity. The consideration of potential side effects is critical in this context. Given FGF21’s indispensable role in maintaining systemic energy homeostasis, global pharmacological inhibition could incur severe metabolic risks, including exacerbated insulin resistance, dyslipidemia, and accelerated hepatic steatosis. Therefore, untargeted systemic blockade of FGF21 is highly contraindicated, reinforcing the necessity for precision tissue-specific interventions. Clinically, FGF21 expression is intrinsically linked to therapeutic responses across multiple modalities. Regarding chemotherapy, FGF21 directly enhances chemoresistance by activating survival pathways (e.g., STAT3/Akt) that mitigate doxorubicin-induced apoptosis. In the context of endocrine therapy, clinical observations indicate that elevated serum FGF21 levels significantly decline following one year of adjuvant endocrine therapy in early breast cancer patients, suggesting a dynamic response to estrogen blockade. Finally, regarding immunotherapy, targeting the FGF21-induced cholesterol overload in CD8+ T cells has demonstrated potent synergistic therapeutic effects when combined with PD-1 blockade, offering a promising strategy to convert immunologically “cold” tumors into “hot” tumors.

Through such precision interpretation and intervention, FGF21 is expected to open promising new avenues for the prevention and treatment of breast cancer, particularly for patients with metabolic abnormality-associated subtypes.

## 7 Conclusion and future perspectives

In summary, this review maintains that FGF21 in breast cancer is far more than a simple metabolic regulator; it represents a pivotal bridge molecule linking systemic metabolic status with local tumor progression. Its functionality is highly context-dependent, contingent upon various factors such as its cellular origin, the metabolic stress within the local microenvironment, and the specific molecular subtype of the tumor. Our perspective is that the functions of FGF21 constitute a unified, rather than a fragmented, whole—its metabolic regulatory and pro-tumorigenic roles are fundamentally two sides of the same biological process, sharing the same underlying molecular pathways. It is precisely this inherent metabolic activity that allows FGF21 to be “hijacked” to support malignant progression when the organism is in a state of metabolic stress, such as obesity or non-alcoholic fatty liver disease (NAFLD).

Consequently, regarding circulating FGF21 levels as an integrative biomarker reflecting the pathological activation of the “metabolism-tumor” axis may offer greater clinical utility than its simplistic classification as a pro-tumorigenic factor. Such an approach could facilitate the identification of specific patient subpopulations whose tumor progression is driven by metabolic dysregulation, thereby enabling the implementation of truly personalized therapeutic strategies.
